# Does source reliability moderate the survival processing effect? The role of linguistic markers as reliability cues

**DOI:** 10.3758/s13421-024-01595-8

**Published:** 2024-06-10

**Authors:** Burcu Arslan, Tilbe Göksun, Çağlar Akçay

**Affiliations:** 1 Department of Psychology, Koç University, Istanbul, Turkey; 2 School of Life Sciences, Anglia Ruskin University, Cambridge, UK

**Keywords:** Evidentiality markers, Source reliability, Survival processing effect

## Abstract

Adaptive memory retains information that would increase survival chances and reproductive success, resulting in the survival processing effect. Less is known about whether the reliability of the information interacts with the survival processing effect. From an adaptive point, information from reliable sources should lead to better encoding of information, particularly in a survival context. In Turkish, specific linguistic components called evidentiality markers encode whether the information presented is firsthand (direct) or not (indirect), providing insight into source reliability. In two experiments, we examined the effect of evidentiality markers on recall across survival and nonsurvival (moving) contexts, predicting that the survival processing effect would be stronger for information marked with evidentiality markers indicating direct information. Results of both experiments yielded a robust survival processing effect, as the sentences processed for their relevance to survival were better remembered than those processed for their relevance to nonsurvival events. Yet the marker type did not affect retention, regardless of being tested as a between- or within-subject factor. Specifically, the survival processing effect persisted even with evidentiality markers indicating indirect information, which suggests that the processing of survival-related information may be privileged even if potentially unreliable. We discuss these results in the context of recent studies of the interaction of language with memory.

## Introduction

Human memory has evolved to enhance people’s ability to solve fitness-relevant problems (Klein et al., 2002; Sherry & Schacter, 1987). Being able to encode and retrieve information that may be relevant to survival is an example of the adaptive value of memory processes. Indeed, a seminal study by Nairne and colleagues (Nairne et al., 2007) showed that information processed for relevance to a survival scenario is better remembered compared with information processed for relevance to a familiar control scenario (moving to a new apartment). This finding termed the *survival processing effect*, suggests that incidentally processing information for its survival value leads to enhanced encoding and subsequent retrieval of that information (Nairne & Pandeirada, 2016). Yet5 such information might be prioritized primarily when it is reliable. When people speak, they frequently provide some linguistic cues indicating source reliability. We ask whether such cues in language affect how survival-related information is retained.

The survival processing effect has since been replicated by many studies, using various control scenarios that vary in their novelty and emotional stimulation (Aslan & Bäuml, 2012; Burns et al., 2013; Kang et al., 2008; Nairne & Pandeirada, 2016; Nairne et al., 2007; Scofield et al., 2018). Thus, there is significant support for the ultimate-level hypothesis that processing information for survival value enhances memory for that information. Several alternative but not mutually exclusive, proximate-level hypotheses have been proposed to explain how this survival processing effect might come about. Some studies focused on the richness of encoding (Kroneisen & Erdfelder, 2011; Kroneisen et al., 2013) or multiple types of processing (e.g., emotional, item-specific, and relational) invoked by the survival context as the proximate mechanism of this effect (Burns et al., 2011). Others emphasized the effects of thinking about the function (Bell et al., 2015) and the concreteness of stimuli (Bell et al., 2013) on later recall.

Another functional hypothesis about human memory is that the memory system should be geared toward encoding and keeping more reliable information, such as information someone acquires through direct experience, compared with less reliable information acquired indirectly through hearsay or gossip (Dancy, 1985; Fitneva, 2008; Giardini et al., 2019). A parallel line of evidence comes from the effect of the perceived reliability of media sources in memory retention, which showed that information from credible sources can have a stronger influence on memory than information from nocredible sources (Ecker & Antonio, 2021; Guillory & Geraci, 2013; Traberg & Linden, 2022). Traberg and Linden (2022) indicated that this pattern was observed even when the political congruency of the source was manipulated, although political tendencies still affected the results.

Language is one of the tools that might provide cues about source reliability. Some languages, like English, encode source information lexically when needed (e.g., with words like *supposedly* or *apparently*). Other languages (e.g., Bulgarian, Korean, Turkish) encode this grammatically via evidentiality markers, indicating whether the information being conveyed is firsthand or not without a need for an additional lexical statement (Aikhenvald, 2004). In the latter group of languages, these evidentiality markers are mostly suffixes added to verbs that indicate whether the information presented in a speech is firsthand or not (Aikhenvald, 2004, 2014; Aksu-Koç, 1988). For instance, in Turkish, evidentiality markers are grammatically encoded in the past tense and mainly differentiate between direct and indirect information (Aksu-Koç, 1988; Aksu-Koç et al., 2009; Slobin & Aksu, 1982). If a verb of a sentence is marked with the -*di* suffix, it means that the information being conveyed is based on firsthand experience (see example 1a below). If a verb of a sentence is marked with the -*miş* suffix, however, the information presented is based on an indirect experience, either hearsay or based on inference (see example 1b below; in the examples, “Sinem” refers to the name of a person).

- (1) a. Sinem toplantıya [katıl-dı].
           Sinem meeting join-PAST. (Conjugated in the past tense with direct evidentiality)
           “Sinem [joined] the meeting” (I saw)

-      b. Sinem toplantıya [katıl-mış].
          Sinem meeting join-PAST. (Conjugated in the past tense with indirect evidentiality)
        “Sinem [joined] the meeting” (I heard/inferred)

Previous research found that evidentiality markers indicating indirect information are preferentially used when lying (Giardini et al., 2019) or when describing inferred as opposed to directly observed events (Ünal et al., 2016). Thus, grammatically encoded evidentially markers have pragmatic implications in terms of providing insight into source reliability (Ünal & Papafragou, 2020).

Consistent with the hypothesis that more reliable information is remembered better, previous studies indicated that marking the source of information in language might influence remembering (Aydın & Fitneva, 2019; Tosun et al., 2013). The effect of evidentiality on memory seems to be particularly prominent in languages where evidentiality is encoded grammatically. For instance, Tosun et al. (2013) found that Turkish speakers showed better recognition and source memory for firsthand information (as marked by the direct evidentiality) about people performing certain activities (e.g., Pınar drank tea after dinner) more than non-firsthand information (as marked by the indirect evidentiality). The same was not the case for monolingual English speakers: lexically marking the source of information (e.g., with *reportedly, presumably, apparently*) in English did not reveal better source memory or recognition for direct than indirect information of the same kind. Similarly, Aydın and Fitneva (2019) presented Turkish adults with sentences, describing situations and actions (e.g., The butterfly entered the kitchen). These sentences were marked with direct or indirect evidentiality. A later free recall task revealed superior retention for the sentences marked with the *-di* than the *-miş* suffix. These findings suggest that obligatorily marking the source of information in a language might lead individuals to prioritize and retain firsthand information, which is more reliable than indirect information.

Studies focusing on listeners’ reliability processing reveal complementary results (Fitneva, 2008; Matsui et al., 2006; Papafragou et al., 2007). For instance, Fitneva (2008) used a judgment task in Bulgarian, a language where the source of information is obligatorily encoded. In this task, children judged sentences containing direct or indirect evidentiality markers and decided which one to believe. They mostly preferred firsthand information over non-firsthand information (but see Öztürk & Papafragou, 2016). Arslan (2020) showed that, while reading, Turkish speakers were quite sensitive to the mismatches between the source of information and the use of evidentiality markers, revealing greater disruptions when the indirect marker was used for firsthand information.

In sum, while human memory seems to be especially able to encode and retain information processed in a survival context, some of this information can be less reliable than others. Grammatically encoding the source of information in a language potentially paves the way for better retention of firsthand information, which in turn may affect the processing of survival-related information as well. Although the survival processing effect has been replicated using various control scenarios, no previous studies asked whether this effect persists when information is less reliable, as signaled by evidentiality markers.

In the current study, we ask whether the survival processing effect depends on the reliability of the information, as indicated by the use of direct versus indirect evidentiality markers. In two experiments, we assessed Turkish speakers’ retention of firsthand and secondhand information marked with evidentiality markers processed in relevance to the survival and nonsurvival contexts. In Experiment 1, participants were shown survival and moving scenarios (within subjects; Nairne et al., 2007). After each scenario, they were presented with short sentences, where the verb was marked with either direct or indirect evidentiality (between subjects). We then measured their retention of presented information. In Experiment 2, we aimed to replicate Experiment 1 by flipping the variables (i.e., making the marker type within subjects and the scenario between subjects).

## Experiment 1

The aim of Experiment 1 was to replicate the survival processing effect and test the role of source reliability in this phenomenon. We manipulated source reliability by using evidentiality markers that are obligatorily encoded in Turkish. Participants were presented with survival and moving scenarios (Nairne et al., 2007) in a counterbalanced order (within subjects). After they read each scenario, they were shown two-word sentences in Turkish (noun + verb) and rated the relevance of each sentence to the scenario they had just read. The verb was the same for all sentences (i.e., *to be*), and it was marked either with direct or indirect evidentiality (between subjects), signaling firsthand and non-firsthand information, respectively. We then tested participants’ retention of words in a surprise recall test.

In line with previous research (e.g., Burns et al., 2013; Nairne & Pandeirada, 2010; Nairne et al., 2007; Weinstein et al., 2008), we expected that information processed for their relevance to the survival context would be better recalled. We also expected that the information marked with the direct marker (*-di* suffix) would be better recalled than the information marked with the indirect marker (*-miş* suffix; Aydin & Fitneva, 2019; Tosun et al., 2013). As direct experiences are more reliable than indirect experiences (Dancy, 1985), and memory systems might be geared toward encoding and retaining more reliable information, this effect might be stronger for the survival context than the moving context, leading to a larger survival processing effect in the direct compared with the indirect information condition. Alternatively, evidentiality markers may not influence the survival processing effect because even if unreliable, potentially survival-related information might be privileged in encoding and storage over nonsurvival information.

### Methods

#### Participants

An a priori power analysis through a G*Power analysis (Faul et al., 2009) indicated the need for 110 participants^1^ for detecting the effect of between-subject effect (evidentiality cues). This sample size exceeds the minimum sample size for detecting only the interaction effect, as explained in Footnote 1. Taking the attrition risk into consideration, we recruited a total of 120 Turkish speakers (74 females) between 18 and 25 years of age (*M*_age_ = 21.39 years, *SD* = 2.47). As we aimed for a medium effect, we set default f as 0.25. Participants were recruited through the subject pool of Koç University and received 0.5 course credit for their participation. The study was approved by the Koç University Ethical Committee on Human Research (2019.319.IRB3.164). We obtained participants’ informed consent before the experiment.

#### Materials

We used the Turkish-translated version (Misirlisoy et al., 2019) of the original survival and moving scenarios of Nairne et al. (2007). We also used two lists of words (List A and List B) that were prepared by Misirlisoy et al. (2019) (Appendix A). Each list consisted of 20 nouns taken from *Turkish Word Norms* (Tekcan & Göz, 2005), and the two lists were matched for frequency, concreteness, and imageability by Misirlisoy et al. (2019).

We prepared the postscenario stimuli by embedding these words into a noun + verb sentence form where the verb was always the same and conjugated in Turkish past tense. The conjugated verbs were *. . . vardı* (2a) or . . . *varmış* (2b), meaning that *there was/were .* *. . *in English (see Appendices B and C for the full sentence lists). All sentences were matched for length, given that they were all two words.

- (2) a. *Mektup* var-dı.
        “There was [a] *letter*” (I saw)

-      b. *Kayısı* var-mış.
        “There was [an] *apricot*” (I heard/inferred)

The scenarios and the sentences were presented on a laptop using the PsychoPy program (Peirce et al., 2019). A blank sheet was provided for the free recall task for participants to write down the words they recalled.

#### Procedure

Experiment 1 was preregistered in Open Science Framework (osf.io/78s2a). We used a mixed design, with the type of evidentiality marker as a between-subjects factor and the scenario type as a within-subject factor. The number of participants per condition was the same. Participants were randomly assigned to the marker-type condition. Some participants were presented with sentences marked with the *-di* suffix (the direct marker condition) and others with the *-miş* suffix (the indirect marker condition; see Appendices B and C). For each scenario, participants were presented with 20 words (Lists A and B). In other words, each participant saw a total of 40 words, 20 for the survival scenario and 20 for the moving scenario. The order of the scenarios, the sentence lists, and the combination of the scenarios and the sentence lists were all counterbalanced.

**Fig. 1:**
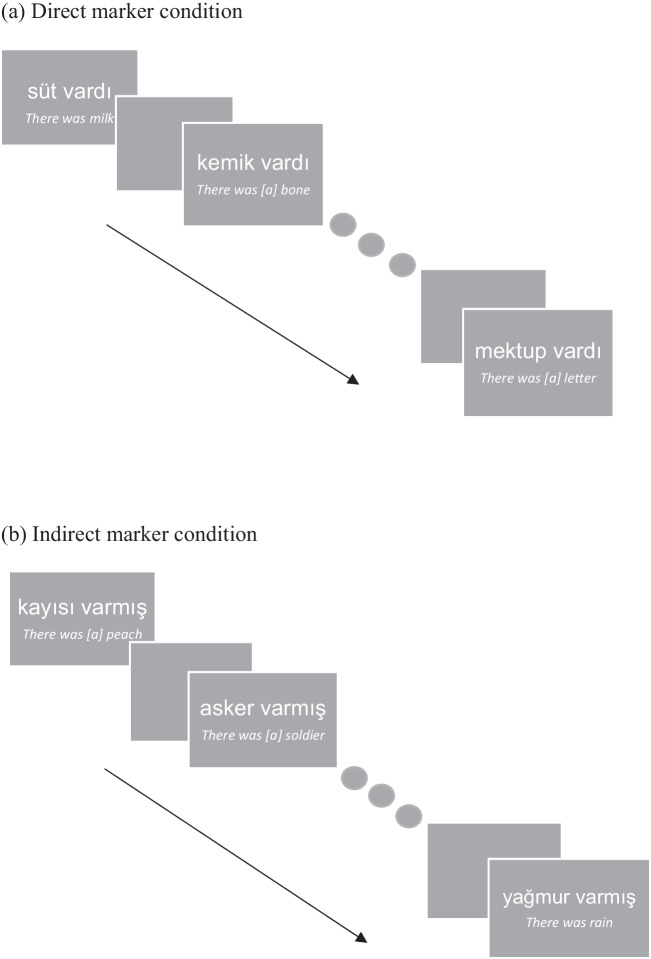
**a–b** Examples from the sentences that were conjugated with the direct (-di) and the indirect (-miş) markers. The English equivalences of the sentences can be seen below the stimuli in italics

We started the experiment by collecting demographic information. Then, participants were seated in front of a computer screen. We explained that the task would be about rating information in relevance to some scenarios, and there would be further instructions in the scenarios to guide them. They rated the relevance of each sentence from 1 (*totally irrelevant*) to 5 (*totally relevant*) separately in relation to the survival and the moving scenarios (note that we did not manipulate the relevancy/irrelevancy of the sentences). The sentences presented after the scenarios were conjugated either with the -*di* (Fig. 1a) or the -*miş* suffix (Fig. 1b), depending on the marker type condition participants were assigned to. The scenarios and the sentences appeared in the middle of a grey screen written in Arial font in white color. Each sentence appeared for 5 seconds and then disappeared. The order of the sentences within each list was also randomized. We recorded both reaction times and ratings during this task with the PsychoPy interface.

After presenting both scenarios, we provided participants with a sudoku task for 3 minutes as a distractor. Then, we gave a surprise recall task to the participants. We asked them to remember the words they just rated and write down the ones they could recall, regardless of any order or referring to scenarios. We told them that they did not need to write the full sentence for each word they remembered. The duration of the free recall task was 10 minutes. In total, the session lasted around 20 minutes. In the end, we asked participants whether they had any guesses about what we intended to measure. None of the participants could guess the purpose of the study. Participants also stated that they did not expect a final memory test.

### Results and discussion

**Fig. 2:**
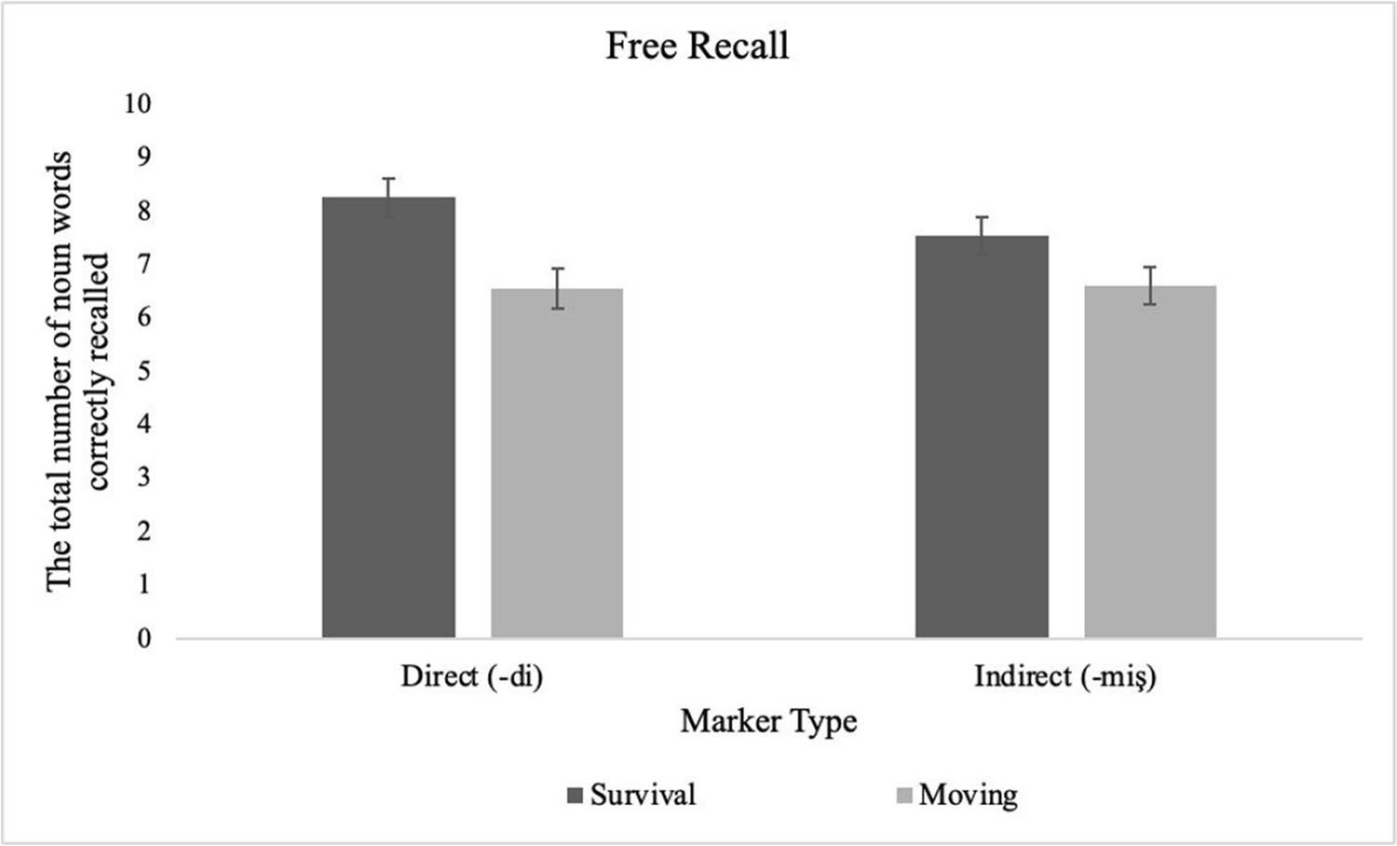
The total number of words correctly recalled for the scenarios across the marker type conditions when the information was given with the direct versus the indirect marker

We conducted a repeated-measures analysis of variance (ANOVA), taking the two scenarios (survival and moving) as the within-subject factor and the type of evidentiality marker (suggesting direct or indirect information) as the between-subjects factor.^2^ The dependent variable was the number of words correctly recalled. Participants recalled significantly more words presented in relation to the survival scenario (*M* = 7.75, *SD* = 2.69) than the moving scenario (*M* = 6.49, *SD* = 2.54), regardless of the type of evidentiality markers—the main effect of scenario: *F*(1, 118) = 23.75, *p* < .001, η_p_^2^ = .168. Note that the recall score for each scenario is out of 20 in this experiment. Since the scenario was the within-subject factor, participants saw a total of 40 words (20 for the survival and 20 for the moving scenario) in the marker type condition they were assigned to. The main effect of marker type, *F*(1, 118) = .95, *p* = .332, η_p_^2^ = .008, and the interaction between scenario and evidentiality was not significant, *F*(1, 118) = 1.13, *p* = .289, η_p_^2^ = .010 (Fig. 2). We also conducted planned comparisons to examine the survival processing effect separately for the direct and indirect markers. Results showed that words processed for their relevance to the survival scenario yielded superior recall relative to the moving scenario both when marked with the *-di* suffix, *t*(59) = 3.92, *p* < .001, *d* = .51, 95% CI [.24, .78], and with the *-miş* suffix, *t*(59) =2.91, *p* = .005, *d* = .38, 95% CI [.11, .64], although the effect size was larger for the *-di* (direct) suffix.

To further investigate the null effect, we carried out a Bayesian analysis using JASP software (Version 0.18.1; JASP Team, 2023). A Bayesian repeated-measures ANOVA showed that the data best supports the model that includes the main effect of the scenario alone (BF_10_ = 4784.856), indicating support for an effect of scenario but little support for the effect of marker type or an interaction effect (Table 1). Consistent with this, the inclusion Bayes factors (BF_incl_; see van Doorn et al., 2021) show very strong evidence for the scenario effect and moderate evidence against both the main effect of marker type and its interaction with scenario (Table 2).

**Table 1:** Model ccomparison of Bayesian repeated-measures ANOVAs to predict the recall score

Models	P(M)	P(M|data)	BF_M_	BF_10_	error %
Null model (incl. subject and random slopes)	0.200	1.488×10^-4^	5.953×10^-4^	1.000	
scenario	0.200	0.712	9.887	4784.856	8.192
scenario + markertype	0.200	0.218	1.118	1468.302	2.244
scenario + markertype + scenario × markertype	0.200	0.069	0.298	466.249	3.370
markertype	0.200	4.464×10^-5^	1.786×10^-4^	0.300	1.284

*Note.* All models include subject and random slopes for all repeated measures factors

**Table 2:** Analysis of effects of Bayesian repeated-measures ANOVAs to predict the recall score

Effects	P(incl)	P(excl)	P(incl|data)	P(excl|data)	BF_incl_
scenario	0.600	0.400	1.000	1.934×10^-4^	3445.866
markertype	0.600	0.400	0.288	0.712	0.270
scenario × markertype	0.200	0.800	0.069	0.931	0.298

We repeated the same frequentist analyses for the average response time and the average relevance rating scores. For the average response times, the main effect of scenario, *F*(1, 118) = 2.92, *p* = .090, η_p_^2^ = .024, the main effect of marker type, *F*(1, 118) = 1.67, *p* = .199, η_p_^2^ = .014, and the interaction between marker type and scenario, *F*(1, 118) = 1.20, *p* = .275, η_p_^2^ = .010, were all nonsignificant. Similarly, for the average rating scores, the main effect of scenario, *F*(1, 118) = 1.19, *p* = .277, η_p_^2^ = .010, the main effect of marker type, *F*(1, 118) = 2.00, *p* = .159, η_p_^2^ = .017, and the interaction between marker type and scenario, *F*(1, 118) = 3.58, *p* = .061, η_p_^2^ = .029, did not yield significant results (Table 3).

**Table 3:** Descriptive statistics of the average response time and the average relevance rating scores

	Average response time (in seconds)				Average relevance rating score			
	Direct marker		Indirect marker		Direct marker		Indirect marker	
Scenarios	*M*	*SD*	*M*	*SD*	*M*	*SD*	*M*	*SD*
Survival	2.99	1.30	2.64	0.94	2.68	0.62	2.92	0.59
Moving	2.70	1.37	2.58	0.89	2.73	0.50	2.74	0.59

*Note. M* = mean, *SD* = standard deviation

The results of Experiment 1 yielded a robust survival processing effect as the words processed for their relevance to the survival scenario were better remembered than those processed for their relevance to the moving scenario, regardless of the evidentiality markers. The effect size of the survival processing effect was slightly larger for the direct than the indirect evidentiality marker, although this difference was not significant (as evidenced by a nonsignificant two-way interaction), and the survival processing effect was nevertheless significant in the indirect evidentiality condition. Therefore, the result of Experiment 1 suggests that the processing of survival-related information may be privileged even if the information is potentially less reliable due to indirect acquisition.

One aspect of Experiment 1, however, may have limited the effect of evidentiality markers. In particular, we manipulated evidentiality as a between-subject factor, and participants only saw either direct or indirect evidentiality markers. Thus, all the information any given participant was presented with was presumably perceived at the same level of reliability (either firsthand and reliable or secondhand and less reliable). When the only kind of information presented is secondhand, people may rely on it as much as they would have relied on firsthand information. In other words, it is possible that the effect of reliability may only be seen in cases where this reliability (as manipulated by evidentiality) varies for a given participant. Therefore, taking the marker type as a within-subject factor instead might lead to an effect of reliability and potentially an interaction of reliability with the survival processing effect. Experiment 2 tests this possibility by presenting evidentiality as a within-subject variable.

## Experiment 2

The aim of Experiment 2 was to replicate Experiment 1 by taking marker type as a within-subject factor and scenario as a between-subject factor. Participants were presented with either survival or moving scenarios, after which they rated the relevance of information marked with direct and indirect markers in a mixed order. We then measured their retention for the words they rated. By taking evidentiality as a within-subject variable, our aim was to increase the power of detecting a difference between the two marker types. Yet we did not prefer a full within-subject design, since we also aimed to replicate the survival processing effect—this time, in a between-subjects design, as Nairne and colleagues (Nairne et al., 2007) did.

### Methods

#### Participants

We recruited 120 Turkish speakers (79 females) between 18 and 25 years of age (*M*_age_ = 20.68 years, *SD* = 1.74). The sample size was the same as in Experiment 1 to make the studies comparable. Participants were recruited through the Koç University Subject Pool and received 0.5 course credit for participating. The experiment was approved by the Koç University Ethical Committee on Human Research (2019.319.IRB3.164). We obtained participants’ informed consent prior to the experiment.

#### Materials

We used the same word lists and design in Experiment 1, except that we changed the design so that the scenario was a between-subject variable and evidentiality was a within-subject variable. The postscenario stimuli were the same as Experiment 1, except that one of the lists was marked with the direct and the other was marked with the indirect marker (see Appendix D for a full list of sentences). We again used the PsychoPy program (Peirce et al., 2019) to present the scenarios and the sentences. As in Experiment 1, participants used a blank sheet to write down the words they recalled in the free recall task.

#### Procedure

The procedure was the same as in Experiment 1, including rating and timing instructions with the exception of the between and within-subjects variables (i.e., the type of evidentiality marker type as a within-subject factor and the scenario type as a between-subject factor). Participants were randomly assigned to one of the scenarios. After being introduced to the scenario, participants were presented with a total of 40 words (List A and List B) in a structured sentence form as in Experiment 1. One of the word lists was presented as marked with the direct marker whereas the other word list was presented as marked with the indirect marker. All participants were presented with both types of markers and the order of the sentences was randomized, regardless of the marker type. The type of marker was intermixed in the sense that participants saw sentences conjugated with the direct or the indirect markers in a mixed and random order. The number of participants per condition was the same. The combination of the markers and the sentence lists were all counterbalanced (see Appendix D, which represents one combination). To control for any bias, participants were not told anything about the evidentiality markers. At the end of the experiment, participants stated that they did not have any specific guess about the purpose of the study, and they did not expect a final memory test.

### Results and discussion

**Fig. 3:**
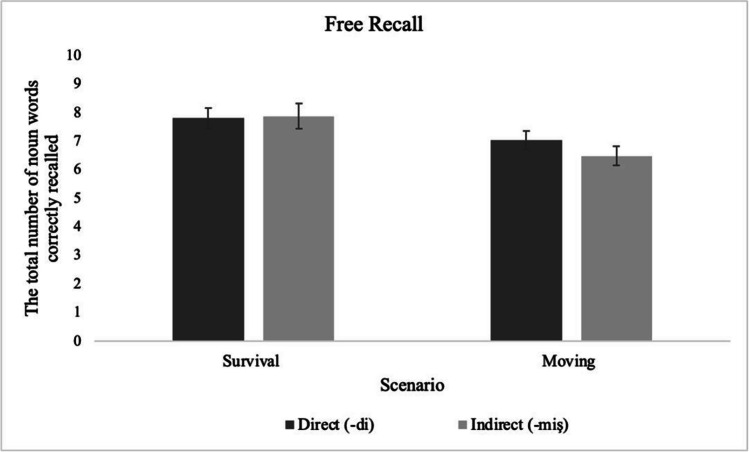
The total number of words correctly recalled for the marker types across the scenarios

We carried out a repeated-measures ANOVA taking the two different scenarios (survival and moving) as the between-subject factor and the marker type (suggesting direct or indirect information) as the within-subject factor. The dependent variable was the number of words correctly recalled. Participants once again recalled significantly more words presented in relation to the survival scenario (*M* = 15.67, *SD* = 5.10)^3^ than the moving scenario (*M* = 13.52, *SD* = 4.33), regardless of the marker type—main effect of scenario: *F*(1, 118) = 6.20, *p* = .014, η_p_^2^ = .050. Note that the recall score for each scenario is out of 40 in Experiment 2 as participants saw 20 words for each marker type, which is the within-subject factor*.* The main effect of marker type, *F*(1, 118) = .67, *p* = .414, η_p_^2^ = .006, and the interaction between scenario and marker type was not significant, *F*(1, 118) = 1.09, *p* = .298, η_p_^2^ = .009 (Fig. 3).

We also conducted a Bayesian analysis using the JASP software, as in Experiment 1. Results of a Bayesian repeated-measures ANOVA showed that the data best supports the model that includes the main effect of the scenario alone (BF_10_ = 3.202), indicating support for an effect of scenario but little support for either the marker type or the interaction of marker type with scenario (Table 4). The inclusion Bayes factors (see van Doorn et al., 2021) show weak to moderate evidence for inclusion the scenario but strong evidence against inclusion of marker type or interaction effect in the models (Table 5).

**Table 4:** Model comparison of Bayesian repeated-measures ANOVAs to predict the recall score

Models	P(M)	P(M|data)	BF_M_	BF_10_	error %
Null model (incl. subject and random slopes)	0.200	0.192	0.953	1.000	
scenario	0.200	0.616	6.419	3.202	2.136
markertype + scenario	0.200	0.118	0.534	0.612	4.984
markertype + scenario + markertype × scenario	0.200	0.037	0.153	0.192	2.876
markertype	0.200	0.037	0.153	0.192	1.683

*Note.* All models include subject and random slopes for all repeated measures factors

**Table 5:** Analysis of effects of Bayesian repeated-measures ANOVAs to predict the recall score

Effects	P(incl)	P(excl)	P(incl|data)	P(excl|data)	BF_incl_
markertype	0.600	0.400	0.192	0.808	0.158
scenario	0.600	0.400	0.771	0.229	2.241
markertype × scenario	0.200	0.800	0.037	0.963	0.153

We then repeated the same frequentist analyses for the average response time and the average relevance rating scores. For the average response times, the main effect of scenario, *F*(1, 118) = .97, *p* = .327, η_p_^2^ = .008, the main effect of marker type, *F*(1, 118) = 1.77, *p* = .186, η_p_^2^ = .015, and the interaction between marker type and scenario, *F*(1, 118) = .92, *p* = .339, η_p_^2^ = .008, were all nonsignificant. Similarly, for the average rating scores, the main effect of scenario, *F*(1, 118) = 2.18, *p* = .142, η_p_^2^ = .018, the main effect of marker type, *F*(1, 118) = 1.13, *p* = .291, η_p_^2^ = .009, and the interaction between marker type and scenario, *F*(1, 118) = .020, *p* = .888, η_p_^2^ < .001, did not yield significant results (Table 6).

**Table 6:** Descriptive statistics of the average response time and the average relevance rating scores

	Average response time (in seconds)				Average relevance rating score			
	Direct marker		Indirect marker		Direct marker		Indirect marker	
Scenarios	*M*	*SD*	*M*	*SD*	*M*	*SD*	*M*	*SD*
Survival	2.55	0.75	2.46	0.97	2.80	0.67	2.76	0.62
Moving	2.42	1.30	2.45	1.42	2.65	0.58	2.60	0.58

*Note. M* = mean, *SD* = standard deviation

Experiment 2 replicated the findings of Experiment 1 by taking marker type as a within-subject factor and scenario type as a between-subject factor. Results again yielded a survival processing effect as participants in the survival scenario condition recalled more words than those who were assigned to the moving scenario condition. Yet we could not find an effect of marker type on retention, although taking marker type as a within-subject factor should have increased power.

## General discussion

In this study, we examined the survival processing effect in relation to Turkish evidentiality markers that provide cues on whether presented information was firsthand or not. We asked whether information marked with the direct marker (*-di*) was remembered better than those marked with the indirect evidentiality marker (*-miş*), especially for the survival context. In two experiments, we showed that words processed for their relevance to the survival context were better retained than words processed for their relevance to the moving scenario. Yet we did not find an effect of evidentiality markers on recall, even when it was included as a within-subject factor in Experiment 2.

Our results are in line with previous research that the survival processing effect is a deeper form of encoding and storing information (Aslan & Bäuml, 2012; Kang et al., 2008; Kroneisen et al., 2013; Nairne & Pandeirada, 2008; Nairne et al., 2007). The reliability cues suggested by grammatical devices such as evidentiality markers might not influence deeper processing like the survival processing effect. In other words, even if a piece of information is suggested to be unreliable with evidentiality markers, information processed for its relevance to the survival context might be privileged in encoding and storage. Indeed, while the modern languages that individuals speak today resulted from a long biological and cultural evolution (Hauser, 1996; Jackendoff, 1999), grammatical evidentiality markers may not affect more fundamental memory processes tapped by the survival processing paradigm. Thus, although source reliability might be an important issue from a functional-evolutionary perspective, providing reliability cues through modern language units might not yield such a profound effect on how individuals process information in survival versus nonsurvival contexts.

These results are also consistent with earlier findings on the effect of survival processing on false memory (e.g., Garner & Howe, 2014; Howe & Derbish, 2010; Otgaar & Smeets, 2010). These studies showed that survival processing might not only enhance recall but also pave the way for false memory illusion (Howe & Derbish, 2010; Otgaar & Smeets, 2010). Similarly, Garner and Howe (2014) showed that the performance in a remote association task was better when participants were primed by false memories, particularly in a survival context compared with a moving context. They argued that survival processing has an adaptive value, which enhances creativity and problem-solving skills regardless of the source of information.

Another interesting point related to our study is that evidentiality markers are obligatorily encoded in the past tense of Turkish, and thus we provided information in the past tense. Klein and colleagues (Klein et al., 2010, 2011) showed that a survival scenario without planning does not lead to a memory advantage, arguing that the key component of the survival scenario is that it is directed toward the future. Yet we found the survival processing effect, although we presented participants with information related to the past, irrespective of whether or not the information was presented as unreliable. More interestingly, the scenarios we used were the Turkish translations of the original moving and survival scenarios (Misirlisoy et al., 2019), which were based on the present tense. It is likely that although information was presented in the past tense, participants assumed that information persisted over time, being somehow linked to the present context. Alternatively, the fact that something is presented in the past tense may not necessarily mean that the item cannot be incorporated in thoughts related to future actions and planning.

There has been very little research on whether language interacts with the survival processing effect. Saraiva and colleagues (Saraiva et al., 2020) found a modest interaction between language and the survival effect. In particular, the survival processing effect existed in the first language (L1) but not in the second language (L2). Their main explanation was based on the higher emotionality evoked in L1 processing (Garrido & Prada, 2021; Harris, 2004). From a functional evolutionary perspective, if the survival processing yielded such a deep effect, one could expect the survival processing effect to persist regardless of the language effect. Indeed, similar to our results, they did not find a significant main effect of language and a significant interaction between language and scenario when they carried out a mixed ANOVA. However, planned comparisons carried out separately to examine the survival processing effect in L1 and L2 found that the survival processing effect was significant in L1 but not in L2, with a larger effect size in L1. In our study, although we found that the survival processing effect was significant for both evidentiality markers, the effect size was larger when the information was presented with direct (*d* = .51) than the indirect marker (*d* = .38). Therefore, Experiment 1 could not rule out a more subtle interaction of reliability processing with survival processing effect. It is nonetheless worth noting that we did not find an overall effect of reliability processing, indicated by the lack of a main effect of marker type on recall. To increase the saliency of reliability cues, we presented evidentiality markers as a within-subject variable in Experiment 2. Although we expected that such manipulation would create a contrast between indirect and direct sources and increase the power, we again could not find an effect of marker type on the survival processing effect.

Earlier work indicates evidentiality markers as having a role in recall and recognition (e.g., Aydın & Fitneva, 2019; Tosun et al., 2013). There is, however, research suggesting that one’s language might not have a strong effect on memory (Papafragou et al., 2007; Ünal et al., 2016). Using photographs that either directly depicted or provided cues about an event, Ünal and colleagues (Ünal et al., 2016) compared Turkish and English speakers’ source memory for those events and found that speakers of both languages were inclined to report the inferred events as they were seen events. Overall, English and Turkish speakers revealed similar patterns, although Turkish obligatorily encodes the source of information in the past tense. Most importantly, unlike Tosun et al. (2013), which found better recognition and source memory for firsthand information in Turkish but not in English, Ünal et al. (2016) used a purely nonlinguistic task to compare Turkish and English speakers’ source memory. The findings of Ünal et al. (2016) are also in line with the study of Papafragou et al. (2007), which found that Korean children did not perform better than their English peers while monitoring the sources of their beliefs, although Korean grammar encodes the source of information. These findings together suggest that mechanisms associated with source monitoring might function similarly across language groups, regardless of evidentiality being obligatorily encoded in grammar. Our results align with these suggestions as we did not find an effect of marking the source of information on the survival processing effect. Even if unreliable, potentially survival-related information was privileged in encoding and storage over nonsurvival information. It is worth noting, however, that information was coming from a neutral source (the computer) rather than a person in this study. It is possible that individuals would retain firsthand and secondhand information differently when information is given by a person.

It is also important to note that our stimuli were slightly different from Tosun et al. (2013) and Aydın and Fitneva (2019), which found an effect of marker type on retention in Turkish. In these studies, they used sentences describing different activities and situations, which were different in content. As a result, they used a different verb for each sentence. Yet, in our study, we used the same verb (i.e., to be) for all sentences, which was marked either with direct or indirect evidentiality, depending on the condition. The words from the lists (Appendix A) that were matched for frequency, concreteness, and imageability were used as subjects to obtain two-word sentences (Appendices B, C, and D). Therefore, our stimuli were more controlled than those of Tosun et al. (2013) and Aydın and Fitneva (2019).

Another aspect is that none of the studies mentioned above required participants to rate the presented information in relation to a scenario. Rating stimuli for relevance to a scenario, as in our case, might evoke context-specific processing along with item-specific processing as a rich form of encoding (Kroneisen & Erdfelder, 2011; Kroneisen et al., 2013). It is possible therefore that the inclusion of markers as source reliability cues had no effect due to the overwhelming effect of deep encoding of rich material. Similarly, in our study, although participants might have an opinion about the reliability of information tagged with direct and indirect markers in the encoding process, mere labeling source reliability via those markers might have had a limited effect on memory, particularly when stimuli were evaluated in relation to a specific context. For instance, Bell and colleagues (Bell et al., 2021) showed that although they provided labels to indicate the level of trustworthiness of information in a given context (e.g., advertisements), those labels did not have a long-term effect on retention. Rather, individuals were more likely to rely on the prior credibility of the information, suggesting a role for schematic effects on memory.

It is worth comparing our study on source reliability with recent studies of the survival processing effect in source memory. Replicating Kroneisen and Bell (2018), Misirlisoy et al. (2019) carried out four experiments in which they compared survival context with nonsurvival contexts (moving and pleasantness) through recall and recognition. They found the survival processing effect both in recall and recognition tasks. In other words, participants remembered more words rated for their relevance to the survival scenario than the moving and the pleasantness contexts. Participants were also asked to indicate the source of the recalled or recognized items as they belong to one of the contexts presented. Source memory was higher for a survival context in all conditions except the free recall task comparing the survival and the moving scenarios (Experiment 1A). In our study, we also used a free recall task after presenting information in relation to survival and moving contexts, but unlike Misirlisoy et al. (2019), after the recall task, we did not ask participants to indicate the words they recalled as they belong to one of the scenarios. Rather, we manipulated source reliability through evidentiality markers while presenting information across survival and moving contexts. Therefore, using the same scenarios and the same experimental stimuli (e.g., word lists) in a free recall task, we extended the findings of Misirlisoy et al. (2019), with a specific focus on source reliability. Although the survival processing effect persisted regardless of the linguistic cues about the source reliability in a free recall task, it is still an open question what the case would be for a recognition task or another control task (e.g., pleasantness).

In conclusion, this study is one of the first that targets language memory interaction concerning the survival processing effect by specifically focusing on language units (i.e., evidentiality markers). Source reliability is critical from a functional evolutionary perspective; however, tools conveying how reliable a source is might yield different effects on the survival processing effect. Focusing on language and its specific units in such contexts would also inform the interaction between language and other cognitive processes.

## Data Availability

The datasets generated during the current study are available in the Open Science Framework repository (osf.io/xv3cm/).

## Appendix A

**Table:**

Word	Translation	List	Frequency	Concreteness	Imageability
ayakkabı	shoe	A	64	6.56	5.99
çerçeve	frame	A	15	6.94	6.47
depo	storage	A	150	6.56	5.57
gözlük	glasses	A	125	6.54	6.08
iskele	pier	A	204	6.74	5.55
kağıt	paper	A	12	6.87	6.36
kan	blood	A	335	6.62	6.27
kemik	bone	A	92	6.88	6.69
lokanta	restaurant	A	69	6.78	6.26
masa	table	A	817	6.86	6.74
mektup	letter	A	50	6.71	5.93
okul	school	A	143	6.63	6.18
salata	salad	A	276	6.93	6.54
şeker	sugar	A	116	6.79	6.42
süt	milk	A	320	6.52	6.11
taşıt	vehicle	A	142	6.69	5.63
terazi	scales	A	10	6.58	6.30
torba	bag	A	408	6.89	6.55
viski	whiskey	A	27	6.83	6.05
yastık	pillow	A	102	6.86	6.71
asker	soldier	B	159	6.82	6.21
bina	building	B	72	6.93	6.44
çömlek	terracotta	B	12	6.85	5.18
damla	drop	B	234	6.60	5.89
ekran	screen	B	323	6.83	6.56
eşya	furnishing	B	73	6.89	6.64
fotoğraf	photograph	B	58	6.51	5.49
heykel	sculpture	B	177	6.67	6.61
kabin	cabin	B	34	6.70	5.59
kablo	cable	B	74	6.86	6.51
kapak	cover	B	81	6.75	6.04
kayısı	apricot	B	17	6.82	6.63
kum	sand	B	73	6.75	6.51
meyve	fruit	B	70	6.79	6.29
parke	parquet	B	16	6.91	5.73
tabure	stool	B	13	6.70	5.88
tahta	wood	B	90	6.87	6.07
televizyon	television	B	164	6.92	6.61
tüy	feather	B	89	6.76	6.14
yağmur	rain	B	57	6.87	6.45

## Appendix B

**Table:**

Sentence	Translation	List
Ayakkabı vardı.	There was [a] shoe. (I saw)	A
Çerçeve vardı.	There was [a] frame. (I saw)	A
Depo vardı.	There was [a] storage. (I saw)	A
Gözlük vardı.	There were glasses. (I saw)	A
İskele vardı.	There was [a] pier. (I saw)	A
Kağıt vardı.	There was [a] paper. (I saw)	A
Kan vardı.	There was blood. (I saw)	A
Kemik vardı.	There was [a] bone. (I saw)	A
Lokanta vardı.	There was [a] restaurant. (I saw)	A
Masa vardı.	There was [a] table. (I saw)	A
Mektup vardı.	There was [a] letter. (I saw)	A
Okul vardı.	There was [a] school. (I saw)	A
Salata vardı.	There was [a] salad. (I saw)	A
Şeker vardı.	There was sugar. (I saw)	A
Süt vardı.	There was milk. (I saw)	A
Taşıt vardı.	There was [a] vehicle. (I saw)	A
Terazi vardı.	There were scales. (I saw)	A
Torba vardı.	There was [a] bag. (I saw)	A
Viski vardı.	There was [a] whiskey. (I saw)	A
Yastık vardı.	There was [a] pillow. (I saw)	A
Asker vardı.	There was [a] soldier. (I saw)	B
Bina vardı.	There was [a] building. (I saw)	B
Çömlek vardı.	There was [a] terracotta. (I saw)	B
Damla vardı.	There was [a] drop. (I saw)	B
Ekran vardı.	There was [a] screen. (I saw)	B
Eşya vardı.	There was furnishing. (I saw)	B
Fotoğraf vardı.	There was [a] photograph. (I saw)	B
Heykel vardı.	There was [a] sculpture. (I saw)	B
Kabin vardı.	There was [a] cabin. (I saw)	B
Kablo vardı.	There was [a] cable. (I saw)	B
Kapak vardı.	There was [a] cover. (I saw)	B
Kayısı vardı.	There was [an] apricot. (I saw)	B
Kum vardı.	There was sand. (I saw)	B
Meyve vardı.	There was [a] fruit. (I saw)	B
Parke vardı.	There was [a] parquet. (I saw)	B
Tabure vardı.	There was [a] stool. (I saw)	B
Tahta vardı.	There was wood. (I saw)	B
Televizyon vardı.	There was [a] television. (I saw)	B
Tüy vardı.	There was [a] feather. (I saw)	B
Yağmur vardı.	There was rain. (I saw)	B

## Appendix C

**Table:**

Sentence	Translation	List
Ayakkabı varmış.	There was [a] shoe. (I heard/inferred)	A
Çerçeve varmış.	There was [a] frame. (I heard/inferred)	A
Depo varmış.	There was [a] storage. (I heard/inferred)	A
Gözlük varmış.	There were glasses. (I heard/inferred)	A
İskele varmış.	There was [a] pier. (I heard/inferred)	A
Kağıt varmış.	There was [a] paper. (I heard/inferred)	A
Kan varmış.	There was blood. (I heard/inferred)	A
Kemik varmış.	There was [a] bone. (I heard/inferred)	A
Lokanta varmış.	There was [a] restaurant. (I heard/inferred)	A
Masa varmış.	There was [a] table. (I heard/inferred)	A
Mektup varmış.	There was [a] letter. (I heard/inferred)	A
Okul varmış.	There was [a] school. (I heard/inferred)	A
Salata varmış.	There was [a] salad. (I heard/inferred)	A
Şeker varmış.	There was sugar. (I heard/inferred)	A
Süt varmış.	There was milk. (I heard/inferred)	A
Taşıt varmış.	There was [a] vehicle. (I heard/inferred)	A
Terazi varmış.	There were scales. (I heard/inferred)	A
Torba varmış.	There was [a] bag. (I heard/inferred)	A
Viski varmış.	There was [a] whiskey. (I heard/inferred)	A
Yastık varmış.	There was [a] pillow. (I heard/inferred)	A
Asker varmış.	There was [a] soldier. (I heard/inferred)	B
Bina varmış.	There was [a] building. (I heard/inferred)	B
Çömlek varmış.	There was [a] terracotta. (I heard/inferred)	B
Damla varmış.	There was [a] drop. (I heard/inferred)	B
Ekran varmış.	There was [a] screen. (I heard/inferred)	B
Eşya varmış.	There was furnishing. (I heard/inferred)	B
Fotoğraf varmış.	There was [a] photograph. (I heard/inferred)	B
Heykel varmış.	There was [a] sculpture. (I heard/inferred)	B
Kabin varmış.	There was [a] cabin. (I heard/inferred)	B
Kablo varmış.	There was [a] cable. (I heard/inferred)	B
Kapak varmış.	There was [a] cover. (I heard/inferred)	B
Kayısı varmış.	There was [an] apricot. (I heard/inferred)	B
Kum varmış.	There was sand. (I heard/inferred)	B
Meyve varmış.	There was [a] fruit. (I heard/inferred)	B
Parke varmış.	There was [a] parquet. (I heard/inferred)	B
Tabure varmış.	There was [a] stool. (I heard/inferred)	B
Tahta varmış.	There was wood. (I heard/inferred)	B
Televizyon varmış.	There was [a] television. (I heard/inferred)	B
Tüy varmış.	There was [a] feather. (I heard/inferred)	B
Yağmur varmış.	There was rain. (I heard/inferred)	B

## Appendix D

**Table:**

Sentence	Translation	List
Ayakkabı vardı.	There was [a] shoe. (I saw)	A
Çerçeve vardı.	There was [a] frame. (I saw)	A
Depo vardı.	There was [a] storage. (I saw)	A
Gözlük vardı.	There were glasses. (I saw)	A
İskele vardı.	There was [a] pier. (I saw)	A
Kağıt vardı.	There was [a] paper. (I saw)	A
Kan vardı.	There was blood. (I saw)	A
Kemik vardı.	There was [a] bone. (I saw)	A
Lokanta vardı.	There was [a] restaurant. (I saw)	A
Masa vardı.	There was [a] table. (I saw)	A
Mektup vardı.	There was [a] letter. (I saw)	A
Okul vardı.	There was [a] school. (I saw)	A
Salata vardı.	There was [a] salad. (I saw)	A
Şeker vardı.	There was sugar. (I saw)	A
Süt vardı.	There was milk. (I saw)	A
Taşıt vardı.	There was [a] vehicle. (I saw)	A
Terazi vardı.	There were scales. (I saw)	A
Torba vardı.	There was [a] bag. (I saw)	A
Viski vardı.	There was [a] whiskey. (I saw)	A
Yastık vardı.	There was [a] pillow. (I saw)	A
Asker varmış.	There was [a] soldier. (I heard/inferred)	B
Bina varmış.	There was [a] building. (I heard/inferred)	B
Çömlek varmış.	There was [a] terracotta. (I heard/inferred)	B
Damla varmış.	There was [a] drop. (I heard/inferred)	B
Ekran varmış.	There was [a] screen. (I heard/inferred)	B
Eşya varmış.	There was furnishing. (I heard/inferred)	B
Fotoğraf varmış.	There was [a] photograph. (I heard/inferred)	B
Heykel varmış.	There was [a] sculpture. (I heard/inferred)	B
Kabin varmış.	There was [a] cabin. (I heard/inferred)	B
Kablo varmış.	There was [a] cable. (I heard/inferred)	B
Kapak varmış.	There was [a] cover. (I heard/inferred)	B
Kayısı varmış.	There was [an] apricot. (I heard/inferred)	B
Kum varmış.	There was sand. (I heard/inferred)	B
Meyve varmış.	There was [a] fruit. (I heard/inferred)	B
Parke varmış.	There was [a] parquet. (I heard/inferred)	B
Tabure varmış.	There was [a] stool. (I heard/inferred)	B
Tahta varmış.	There was wood. (I heard/inferred)	B
Televizyon varmış.	There was [a] television. (I heard/inferred)	B
Tüy varmış.	There was [a] feather. (I heard/inferred)	B
Yağmur varmış.	There was rain. (I heard/inferred)	B

## Footnotes

1 We determined the sample size (110 plus an additional 10% for the risk of participant attrition) through a G*Power analysis (Faul et al., 2009). We set the test family as *F tests*, the statistical test as *ANOVA: Repeated measures, between-factors*, the type of power analysis as *a priori*, *effect size f* as 0.25, *power* as 0.85, *number of groups* as 2, and *number of measurements* as 2. Please note that the statistical test was set as *ANOVA: Repeated measures, between-factors* since we were concerned with the effect of evidentiality markers as the between subjects factor in Experiment 1. We acknowledge that because our main hypothesis dealt with the interaction effect of survival and evidentiality, setting the statistical test as *ANOVA: Repeated measures, within–between interaction* would also have been valid. The latter option using the same input parameters would require the sample size to be 38 instead. Thus, determining the sample size with the between-subjects factor in mind led to an overpowered sample for detecting the interaction effect.

2 We carried out the same ANOVA by also including the scenario order as a between-subjects variable. Results showed that the main effect of the order was not significant, *F*(1, 116) = 2.84, *p* = .094, η_p_^2^ = .024. The interaction between scenario and order, evidentiality and order, as well as scenario, evidentiality, and order, were not significant, *F*(1, 116) = 2.31, *p* = .131, η_p_^2^ = .020; *F*(1, 116) = .021, *p* = .884, η_p_^2^ = .000; and *F*(1, 116) = .177, *p* = .675, η_p_^2^ = .002, respectively. Therefore, we did not include the presentation order of scenarios in the main analyses.

3 These mean values in Experiment 2 belong to the main effect of the scenario as a between-subjects factor (the sum of recalled words with direct and indirect marker types for each scenario). In Fig. 3, we presented the recall scores for each marker type separately.
